# Reliability of hand-held dynamometer in measuring gluteus medius isometric muscle strength in healthy population

**DOI:** 10.12669/pjms.38.5.5207

**Published:** 2022

**Authors:** Rabail Rani Soomro, Hossein Karimi, Syed Amir Gillani

**Affiliations:** 1 Dr. Rabail Rani Soomro, PT, Assistant Professor, Sindh Institute of Physical Medicine and Rehabilitation, Karachi, Pakistan; 2 Hossein Karimi, PT, PhD. Professor Istanbul Gelisim University, Turkey. Adjunct Professor, University Institute of Physiotherapy, University of Lahore, Lahore, Pakistan; 3 Prof. Dr. Syed Amir Gillani, PhD. Professor & Dean, Faculty of Allied Health Sciences, University Institute of Physiotherapy, University of Lahore, Lahore, Pakistan

**Keywords:** Reliability, Hand-held dynamometer, Isometric strength

## Abstract

**Objectives:**

To determine the inter-rater and intra-rater reliability of the hand-held dynamometer in measuring isometric gluteus medius muscle strength in asymptomatic healthy population.

**Methods:**

It was a cross sectional study conducted at the department of physiotherapy, Sindh Institute of Physical Medicine and Rehabilitation from March 2021 to April 2021. Thirty healthy subjects aged 18 to 25 years of both sexes and no previous or current complaints of hip or knee pain were included through non-probability purposive sampling technique. The strength of the unilateral isometric gluteus medius muscle was measured using a hand-held dynamometer by two raters at the same day and a week later. Pearson Correlation coefficient was calculated to see the relationship of muscle strength measured by each rater within and between day’s sessions. Intra–class correlation was calculated with 95% confidence interval and Standard error of measurements using reliability analysis.

**Results:**

In the present study there were thirty participants studied by two raters, the mean age of participants was 21.53 (SD=±1.40) years, the mean BMI was 24.05 (SD=±1.12) kg/m2 and 70% participants were female and 30% were males. This study showed the mean muscle measurement of participants within days was 12.92 (SD=±0.94), with intra-class correlation ICC (2,2) 0.94 and SEM 0.12 and mean muscle measurement of patients between days was 12.99 (SD=±0.91), with intra-class correlation ICC (2,2) 0.90 and SEM 0.12..

**Conclusion:**

Hand-held dynamometer has shown excellent inter-rater and intra-rater reliability in measuring isometric strength of Gluteus Medius muscle among healthy population. It is convenient to be used in clinical settings and can be a useful outcome tool to assess strength in interventional studies.

## INTRODUCTION

The assessment of a muscle strength through a reliable method is considered as an essential component of physical examination. In Pakistan and worldwide, routine practice to measure muscle strength clinically is done by a procedure of Manual Muscle Testing (MMT). MMT regardless of its usage as a current standard of practice, has demonstrated several limitations in the latest literature.^1^ It has been shown to be inadequate in measuring the muscles of lower extremity with larger size and greater force.^2^ Hip biomechanics plays a vital role in physical performance and day to day physical activity. Being a multi-pennate muscle, Gluteus Medius (Gmed) is the primary hip abductor that provides the most strength for hip abduction.^3^ Weakness of Gmed results in compensatory motion of the lower back, hip, and knee.^4^ Consequently, Gluteus Medius strengthening may be crucial not only for rehabilitation but also injury prevention. Sufficient understanding of Gmed weakness is required to expedite optimum hip function or design customized Gmed strengthening exercise for individuals with Gmed weakness.^5^

Several methods exist for estimating strength of the hip abductors. A much debate exists over the reliability of Manual Muscle Testing (MMT) grades, as the visual and palpations skills are examiner dependent that are based on their experiences.^6^ For better accuracy in clinical examination the devices such as hand-held dynamometer (HHD) and isokinetic machines are considered as the valid and reliable equipment’s. Isokinetic machine despite being the gold standard, is highly expensive and time consuming equipment which is why it is not readily accessible for most practitioners.^7^ On the other hand, HHD can be used as an alternative tool to isokinetic machine as they are time sufficient, portable and relatively low-cost method of measuring strength.^8^ Another advantages of HHD include a quick tool of providing objective values in clinic and experimental settings.^9^ The HHD can be used to quantify hip strength; however, reliability of the device to gain the accurate results particularly with the Gluteus Medius muscle remains unclear. Therefore, the purpose of this study was to assess the inter-rater and intra-rater reliability of the hand-held dynamometer in measuring Gluteus Medius isometric muscle strength in asymptomatic healthy population.

## METHODS

This cross-sectional study was part of PhD physical therapy project following the approval from Institutional Review Board (IRB) of University of Lahore. Study was conducted from March 2021 to April 2021 by using a non-probability purposive sampling technique at Sindh Institute of Physical Medicine and Rehabilitation. The expected ICC =0.75 for excellent rating at 95% confidence interval and 90% power 2 30 sample size was used in this study. Thirty healthy subjects (21 female and 9 male) aged 18 to 25 years with no previous or current complaints of hip or knee pain participated in this study after giving written informed consent. The strength of the gluteus medius muscle was assessed on the dominant side using the Microfet2 hand-held dynamometer (MicroFET 2, Hoogan Health Industries, West Jordan, UT, USA). With the subjects in a side-lying position, a pillow was placed between the knees of the participants, the hip of the testing leg was approximately 10^0^ abductions. The MicroFET-2 hand-held dynamometer was placed 5.0cm proximal to the lateral knee joint line. The subjects were instructed to push the thigh upwards with maximal effort for five seconds. For maximum abduction force, the peak output of the three trials was used. Two physiotherapists with sufficient clinical experience and trained in using dynamometer were responsible for the collection of data. Both raters and subjects were unaware of the findings and results. In the inter-rater’s step, both raters did the assessment with an interval of an hour on the same day. While in intra-rater’s step both raters did the assessment a week later following the 1^st^ assessment.

Data were stored and analyzed using IBM-SPSS version 23.0, mean and standard deviation for muscle strength were reported from two raters at three sessions; the mean comparison of muscle strength between and within days for each rater was done using paired sample-test, and between raters was done using independent sample-test. Pearson Correlation coefficient was also reported to see the relationship of muscle strengths of raters within and between day’s sessions. Intra-class correlation was also reported with 95% confidence interval and Standard error of measurements using reliability analysis. P-values less than 0.05 were considered statistically significant.

## RESULTS

In the present study there were thirty participants studied by two raters, the mean age of participants was 21.53 (SD=±1.40) years, with range values 19 – 24, the mean body mass index was 24.05 (SD=±1.12) kg/m^2^ with range value 22 - 25.8. In this study 70% participants were female and 30% were males. Table-I reports the descriptive data on muscle strength by two different raters in three sessions of study.

**Table I:** Descriptive Statistics on Muscle Strength kgf (n=30).

Raters and Sessions	Mean	SD	Range	Min.	Max.
R1 Session-I	13.02	1.06	3.80	10.90	14.70
R1 session-II	12.90	0.93	3.70	10.80	14.50
R1 session -III	13.08	0.86	3.30	11.00	14.30
R2 Session-I	12.84	0.94	3.20	11.00	14.20
R2 session-II	12.94	0.89	3.40	11.00	14.40
R2 session -III	13.03	0.89	3.60	11.00	14.60

SD: Standard Deviation, R1: Rater-I, R2: Rater-II.

There were 96% positive correlation for muscle strength within days and 92% positive correlation for muscle strength between days of Rater-I correlation, it also reports there were 94% positive correlation for muscle strength within days and 91% positive correlation for muscle strength between days of Rater-II correlations. Table-II The paired sample t-test showed a significant mean difference in muscle strength for within days and between days with p-value less than 0.05.

**Table II:** Inter Rater Pair-wise Comparison of Muscle Strength.

Muscle Strength	Raters	r-value (p-value)	t-value (p-value)
Within Days	R1	0.96 (<0.01*)	2.17 (0.038*)
	R2	0.94 (<0.01*)	-1.73 (0.093)
Between Days	R1	0.92 (<0.01*)	-0.85 (0.398)
	R2	0.91 (<0.01*)	-2.65 (0.013*)

r: correlation Coefficient, t-value: Paired Sample t-test

* p<0.05 was considered statistically Significant.

The mean comparison of Rater-I and Rater-II for muscle strength is shown in Table-V. Independent sample-test showed there was no significant mean difference in mean muscle strength of Rater-I and Rater-II for within days and between days sessions.

**Table III:** Mean ± SD and ICC (2, 1) for within day and between day’s intra-rater reliability measures of Muscle Strength.

Reliability	Rater	Mean SD	ICC (95% C.I)	SEM (deg)
Within Days	R1	12.9±0.98	0.95* (0.90 – 0.97)	0.18
	R2	12.8±0.90	0.94* (0.88 – 0.97)	0.16
Between Days	R1	13.0±0.94	0.90* (0.80 – 0.95)	0.17
	R2	12.9±0.9	0.91* (0.82 – 0.95)	0.16

ICC (2,1): Intra-class Correlation Coefficient; SEM: Standard Error of Measurement.

Deg (Degree) R1: Rater-I, R2: Rater-II,

* P<0.05 considered statistically significant

**Table IV:** Mean ± SD and ICC (2, 2) for within day and between day’s inter-rater reliability measures of Muscle Strength.

Reliability	Mean± SD	ICC (95% C.I)	SEM (deg)
Within Days	12.92±0.94	0.94* (0.90 – 0.96)	0.12
Between Days	12.99±0.91	0.90* (0.84 – 0.94)	0.12

ICC (2,2): Intra-class Correlation Coefficient; SEM: Standard Error of Measurement;

Deg (Degree) R1: Rater-I, R2: Rater-II,

* P<0.05 considered statistically significant

**Table V:** Comparison of Mean Muscle Strength between Two Raters.

Sources	Raters	N	Mean	SD	p-value
Within Days	R1	30	12.95	0.98	0.79
	R2	30	12.89	0.90	
Between Days	R1	30	13.05	0.94	0.63
	R2	30	12.93	0.89	

*p<0.05 was considered statistically significant using Independent Sample t-test.

## DISCUSSION

This cross-sectional study verifies that the HHD has excellent inter-rater and intra-rater reliability. Current study results suggest that there were 96% (Rater-I) and 94% (Rater-II) positive correlation for muscle strength assessed within days, and 92% (Rater-I) and 91% (Rater-II) positive correlation for muscle strength assessed between days which is found statistically significant with p<0.01. To the authors knowledge, this study is first of its kind to perform the inter-rater and intra-rater pairwise comparison and correlation to determine the strength of gluteus medius muscle using HHD. This study was done prior to experimental study of PhD project hence the study results can be helpful that confirm that the dynamometer can be widely used as an outcome measurement tool to assess the hip abduction muscle strength in Interventional studies. In present study, inter-rater reliability for MicroFet2 HHD in healthy population within days was 0.94 and between days was 0.90. A study by Awwad DH et al showed the intra-assessor and inter-assessor ICCs for testing hip abductor muscle strength using MicroFET 2 HHD were both high at 0.92 to 0.94.^10^ Although the difference lies in the chosen population, current study involved healthy young population while the previous study involves older population. Similarly, another very recent study on young athletes determined the intra-examiner reliability for the GMed Clinical Test with MicroFET 2 dynamometer very high (Examiner 1=0.98; Examiner 2=0.96), and the inter-rater reliability for the GM Clinical Test was also considered high (0.95)^11^ which is closely aligned with current study results that also shows high intra-rater reliability for within days as (Rater 1= 0.95; Rater 2=0.94). A study by Romero-Franco N et al on healthy participants concluded that the intra-tester reliability (ICC > 0.88; SEM < 16.3; P < 0.001) and the inter-tester reliability (ICC > 0.83, SEM < 11.9, P ≤ 0.002) was almost perfect for all the lower limb movements except for hip abduction, that was substantial (ICC = 0.764, SEM = 7.39, P = 0.008).^12^ Contrary to our study where the inter-rater reliability remained excellent (ICC=0.94 for within days and ICC= 0.90 for between days) with p value <0.05.

The magnitude of computed ICCs remains to be consistently excellent and does not show much variability in different populations assessed. Supporting the statement another latest study done on professional dancers concluded that HHD can reliably measure force production of hip, knee, and ankle muscle groups without use of external fixation devices.^13^ Similar to previous study current study also did not use any external fixation device, and the measurements were highly dependent on the skill and strength of the assessor. However, some studies have discouraged this idea and encouraged the use of externally fixed dynamometers to minimize the influence of assessor.^14^-^16^

The ICCs reported in current study are like previous studies that have reported the intratester reliability of side-lying hip abduction with a dynamometer to be ranged between 0.76 and 0.98.^16^-^18^ Additionally these studies also supported the side-lying position to be the best position to get the maximum hip abduction force as performed with subjects in the present study. However fewer studies among these asserted the range of motion of testing leg to be in neutral which is contrary to current study because the starting position of testing leg used by the physiotherapists was 10^0^ abductions. Moreover, the testing leg used in the current study was a dominant side. Nevertheless, a recent study shows that no significant difference was seen in the clinical hip abductor strength measurements with the dynamometer between dominant and non-dominant sides.^19^ In the present study mean muscle strength was also assessed between two raters which showed no significant difference in the extent of measurement of strength between two raters for between days and within days measurements. It could be probably because both raters were experienced and qualified physiotherapist. Previous study found the differences in the mean measurements taken by the experienced physiotherapist, novice physiotherapist and student physiotherapist.^13^ Another latest study by Fenato RR found the relation of gluteus medius muscle strength with obesity and normal weight. 20 In the current study BMI was calculated for each participant, but no correlation of BMI was explored with the muscle strength assessed.

### Limitations of the study:

The study was done on asymptomatic healthy population so the results cannot be extrapolated to other population. Secondly in this study only side lying position was used due to time constraint.

## CONCLUSION

The MicroFET 2 HHD shows excellent inter-rater and intra-rater reliability in assessing isometric muscle strength of Gluteus Medius. Its accurate objective values make it a valuable tool to be used in clinical trials and clinical settings.

### Authors’ Contribution:

**RRS, HK & SAG:** Conceived, designed, and performed Statistical analysis and are accountable for accuracy and integrity of work.

**RRS, HK:** Did data collection, manuscript writing and editing.

**HK, SAG:** Did review and final approval of manuscript..
